# 2459 Hippocampal network disruption in early amyloid pathology

**DOI:** 10.1017/cts.2018.81

**Published:** 2018-11-21

**Authors:** Adam Caccavano, Stefano Vicini

**Affiliations:** Georgetown – Howard Universities

## Abstract

OBJECTIVES/SPECIFIC AIMS: We aim to show that amyloid accumulation in an animal model of Alzheimer’s disease leads to a preferential disruption of inhibitory parvalbumin-expressing interneurons, and the peri-neuronal nets that surround them, resulting in downstream network alterations to potentially explain early mechanisms of memory impairment in the disease. METHODS/STUDY POPULATION: We employ the 5xFAD mouse model of familial Alzheimer’s disease crossed with transgenic mouse lines which fluoresce red or green in specific neuronal populations. We conducted immunostaining and immunoblotting in amyloid accumulating animals compared with healthy littermate control. Future experiments will be performed in human postmortem tissue to translate these results from mouse model to the human population. Electrophysiological recordings from acute mouse brain slices were conducted as a functional assay. RESULTS/ANTICIPATED RESULTS: Preliminary results indicate that PNNs are disrupted and that activity-associated levels of PV are reduced. Both inhibitory PV and excitatory pyramidal cell populations exhibit altered spiking and synaptic activity during sharp wave ripple events. DISCUSSION/SIGNIFICANCE OF IMPACT: By elucidating the specific neuronal sub-type that is responsible for hippocampal network disruption, future studies could attempt a targeted optogenetic or pharmacological intervention to restore network activity important for memory consolidation.

